# Anti-Swelling Hydrogel Wearable Sensors: Structural Engineering, Internal Water Environment Regulation, and Motion Monitoring in Complex Environments

**DOI:** 10.3390/gels12070639

**Published:** 2026-07-17

**Authors:** Qinglei Li, Ping Shen, Zhihao Liu, Haonan He, Weiquan Shi, Hao Hong, Jaeyoung Park, Kaixin Xu, Jie Wu

**Affiliations:** 1 College of Art and Physical Education, Kyungil University, 50, Gamasil-gil, Hayang-eup, Gyeongsan-si 38428, Gyeongbuk-do, Republic of Korea; 20259132@kiu.kr (Q.L.); 18809800712@163.com (H.H.); 15978787319@163.com (W.S.); sports@kiu.kr (J.P.); 2 School of Physical Education, Anshun University, Anshun 561000, China; 3 School of Physical Education, Hubei University of Automotive Technology, 167 Checheng West Road, Shiyan 442002, China; liuzhihao92@163.com; 4 School of Wushu, Henan University, Kaifeng 475001, China

**Keywords:** anti-swelling hydrogels, wearable sensors, water transport, internal water environment, motion monitoring

## Abstract

As wearable sensors advance toward long-term motion monitoring and operation in humid environments, performance priorities are shifting from sensitivity to sustained reliability. Hydrogels are attractive sensing materials due to their tissue-like compliance, biocompatibility, and tunable conductivity; however, their hydrated networks readily absorb water under perspiration, high humidity, and underwater conditions, leading to structural relaxation, interfacial instability, conductive pathway disruption, and signal drift. Thus, anti-swelling design should move beyond reducing swelling ratios toward coordinated regulation of water transport, internal water environment, interfacial integrity, and signal stability. This review summarizes recent advances in anti-swelling hydrogel-based wearable sensors, focusing on structural engineering strategies, including network confinement, surface hydrophobicity, core–shell architectures, and gradient structures, as well as material regulation mechanisms, including ionic/coordination crosslinking, nanoconfinement, zwitterionic hydration, and solvation-mediated anti-water exchange, highlighting their synergistic roles in long-term anti-swelling performance and environmental adaptability. Representative applications in perspiration monitoring, underwater motion sensing, rehabilitation, and intelligent interaction demonstrate the importance of anti-swelling regulation for reliable sensing in wet environments. Finally, the remaining challenges are summarized, together with future perspectives on the synergistic design of structures, materials, and interfaces, standardized evaluation systems for realistic motion environments, and scalable manufacturing. Anti-swelling hydrogel sensors are expected to evolve from low-swelling materials into environmentally adaptive sensing platforms for aqueous environments, enabling advances in underwater sports monitoring, digital health, and underwater human–machine interaction.

## 1. Introduction

With the rapid advancement of flexible electronics, digital healthcare, and intelligent sports monitoring technologies, wearable sensors are transitioning from short-term signal acquisition platforms to systems capable of long-term continuous monitoring and stable operation under complex environmental conditions. Compared with conventional rigid sensors, flexible wearable devices must maintain conformal contact with dynamic biological interfaces—including skin, joints, muscles, and moving body surfaces—while delivering reliable signal output under repeated bending, stretching, friction, and sweat exposure [1,2,3]. Consequently, the performance evaluation of wearable sensing systems has extended beyond traditional metrics such as sensitivity, detection range, and response speed to emphasize long-term operational stability, interfacial adaptability, and signal reliability under practical conditions. This shift is particularly critical in high-humidity and perspiration-rich environments [4], underwater training [5], rehabilitation exercise [6], and humid–thermal monitoring scenarios [7], where sensing materials experience not only sustained mechanical deformation but also prolonged exposure to moisture [8], ionic media [9], fluid shear and flushing [10], and interfacial hydration effects [11]. These coupled environmental stresses impose increasingly stringent requirements on sensor structural integrity and long-term signal stability.

Hydrogels have emerged as an important material platform for wearable sensors owing to their tissue-like softness, biocompatibility, tunable conductivity, and favorable interfacial compatibility with human skin [12,13,14]. Their three-dimensional hydrated network enables effective buffering of mechanical mismatch generated during body movement and supports the detection of strain, pressure, electrophysiological, and environmental signals through ionic conduction, electronic conduction, or hybrid conductive mechanisms [15,16]. Nevertheless, these advantages are accompanied by inherent limitations. Due to the abundance of hydrophilic functional groups and free water within the polymer network, hydrogels are highly susceptible to continuous water uptake and swelling under humid, perspiration-rich, and underwater conditions. The swelling process can induce polymer chain expansion [17], enlargement of network pore size [18], degradation of mechanical properties [19], and redistribution of conductive components [20], which subsequently results in interfacial delamination, interfacial impedance drift, disruption of conductive pathways, and instability of signal output. For wearable sensing systems, these changes represent not merely dimensional variations but a direct threat to the accuracy and reliability of long-term continuous monitoring.

Accordingly, the development of anti-swelling hydrogels has become an important strategy for enhancing the long-term operational performance of wearable sensors in humid environments. It should be noted that anti-swelling capability does not simply refer to reducing water absorption or pursuing the lowest possible swelling ratio. Although strategies such as increasing crosslinking density, reinforcing hydrophobic barriers, or constructing highly constrained network architectures can effectively inhibit volume expansion, these approaches may also lead to increased material rigidity, hinder ion transport, reduce interfacial adhesion, and impair wearing comfort [21]. For wearable sensing applications, effective anti-swelling design should not be limited to maintaining dimensional stability, but should instead realize synergistic regulation of water transport behavior, free and bound water distribution, network structural integrity, conductive pathway retention, and stable signal output, while preserving mechanical flexibility, electrical conductivity, and interfacial adaptability [22]. Fundamentally, the goal of anti-swelling engineering is not merely to prevent hydrogel swelling, but to ensure that wearable sensing systems can maintain continuous, stable, and long-term operation under complex humid conditions.

In recent years, considerable efforts have been devoted to developing anti-swelling hydrogels through various design strategies. From the perspective of structural design, methods including network confinement [23], surface hydrophobization [3], core–shell structures [24], and gradient architectures [25] have been adopted to improve dimensional stability by limiting polymer chain expansion, slowing water diffusion, constructing interfacial barriers, and optimizing stress distribution. At the same time, material strategies based on anti-swelling mechanisms, such as ionic/coordination crosslinking [26], nanoscale confinement [27], zwitterionic hydration regulation [28], and solvation-mediated inhibition of water exchange [29], further regulate the internal water environment by adjusting the balance between free water and bound water, weakening the driving force of water exchange, and maintaining the continuity of conductive networks. These strategies synergistically suppress swelling through the combined effects of structural engineering and material regulation, thereby providing a foundation for long-term stable monitoring under complex environments. Nevertheless, current studies still primarily evaluate anti-swelling performance using macroscopic metrics, such as equilibrium swelling ratio, mass variation, and mechanical retention, while a systematic understanding of water transport behavior, the dynamic evolution of free and bound water, and their intrinsic relationship with long-term signal stability remains lacking. Meanwhile, several recent reviews have summarized advances in wearable hydrogel sensors, anti-swelling hydrogels, and organohydrogels, primarily focusing on material composition, anti-swelling strategies, or sensing applications. However, few have systematically established the relationship among anti-swelling mechanisms, internal water environment regulation, adaptation to complex environments, and long-term motion monitoring from the perspective of practical wearable application requirements.

Based on these considerations, this review takes “structural design–internal water environment regulation–adaptation to complex environments–motion-monitoring applications” as its central framework to systematically summarize the structural design strategies and internal water environment regulation mechanisms of anti-swelling hydrogels. Particular emphasis is placed on elucidating the influence of anti-swelling properties on interfacial stability, conductivity retention, and long-term signal reliability. Furthermore, by focusing on two representative motion environments, namely high-humidity perspiration and underwater conditions, this review summarizes the key evaluation metrics for different application scenarios and further proposes an evaluation framework for practical wearable applications, thereby providing a reference for the rational design and engineering translation of anti-swelling hydrogel-based wearable sensors.

## 2. Structural Strategies for Anti-Swelling Hydrogel-Based Wearable Sensors

Hydrogels inevitably undergo water uptake and swelling during long-term service, primarily as a consequence of osmotic pressure generated by external water infiltration, accompanied by polymer chain expansion and volumetric enlargement of the network. For wearable sensors, swelling not only induces dimensional deformation but also weakens interfacial adhesion, reconstructs conductive pathways, and causes signal drift, thereby compromising the long-term operational stability of the device under complex environmental conditions. Therefore, the primary objective of structural design is not merely to increase crosslinking density, but rather to regulate water transport and maintain network stability by retarding water diffusion, restricting polymer chain mobility, and optimizing stress distribution under hydrated conditions, thereby synergistically enhancing anti-swelling capability and sensing stability.

Current structural anti-swelling strategies can be broadly categorized according to the spatial hierarchy at which water transport is regulated, including bulk network confinement [30,31], interfacial water-barrier engineering [32,33], spatial functional partitioning [34,35], and continuous gradient regulation [36,37]. Based on the location and spatial organization of these structural features, these strategies are further classified in this review into four representative architectures: network-constrained structures, surface-hydrophobic structures, core–shell structures, and gradient structures. These four architectures correspond to distinct levels of water transport regulation, ranging from bulk network confinement and interfacial barrier formation to spatial compartmentalization and continuous gradient modulation. Collectively, they encompass the major design paradigms of current structural anti-swelling hydrogels while providing a unified spatial regulation perspective for understanding the intrinsic design logic underlying different structural configurations. Nevertheless, each architecture presents distinct advantages and limitations, requiring careful trade-offs among anti-swelling capability, mechanical robustness, conductivity, fabrication complexity, and practical applicability (see Table 1).

### 2.1. Network-Confinement Structures

Network confinement is the most fundamental structural strategy for anti-swelling hydrogels, functioning by suppressing water-induced volumetric expansion through enhanced network stability, reduced free volume, and restricted polymer chain extension [48]. Early studies primarily relied on single-network crosslinking or increased crosslinking density to improve dimensional stability, but excessive confinement often compromised flexibility, stretchability, and sensing performance. To address these limitations, recent efforts have evolved from conventional single-network structures [49] toward advanced architectures, including double-network systems [38,50], multiple-network structures [51,52], interpenetrating polymer networks [53,54,55], and chain-entangled networks [56,57]. These designs improve structural stability and anti-swelling performance through multiscale synergistic reinforcement while reducing the trade-offs associated with excessive network constraint [58].

**Figure 1:**
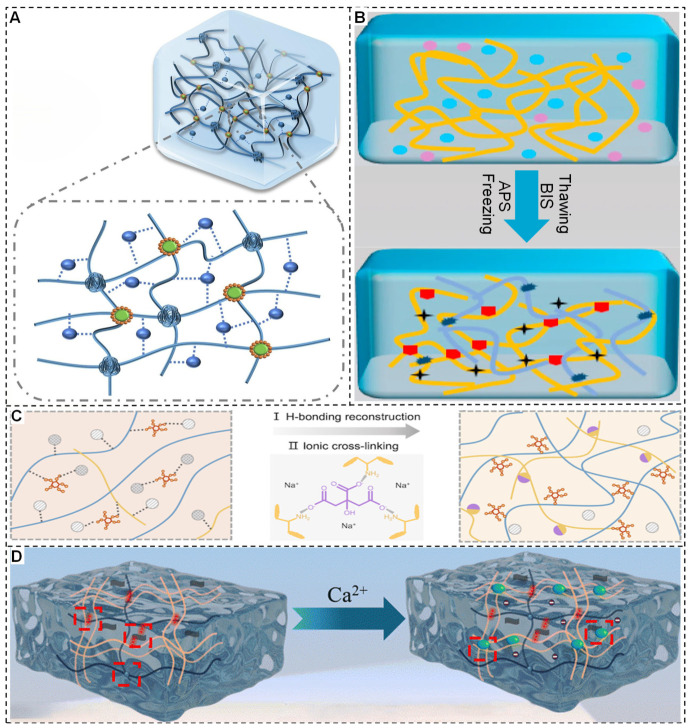
Network architecture–enabled design strategies for anti-swelling hydrogels. (**A**) Anti-swelling hydrogel design based on a single-network architecture reinforced by multiple physical crosslinking interactions [59]. (**B**) Anti-swelling hydrogel design based on a DN architecture [22]. (**C**) Anti-swelling hydrogel design based on a multi-network architecture [61]. (**D**) Anti-swelling hydrogel design based on a semi-interpenetrating polymer network architecture [8].

Single-network structures represent the most straightforward strategy for constructing anti-swelling hydrogels, primarily relying on increased crosslink density or multiple physical interactions to stabilize the network. For example, Liu et al. [59] developed a physically reinforced single-network hydrogel based on a methacrylamide/2-hydroxyethyl methacrylate (HEMA) copolymer network (Figure 1A). The synergistic effects of SiO_2_ nanoparticles, hydrophobic micelles, and hydrogen bonding formed a stable framework, enabling the hydrogel to maintain structural integrity after 67 days of continuous immersion. However, the anti-swelling performance of single-network systems remains inherently limited, and excessive crosslinking often sacrifices toughness and deformability. To overcome these drawbacks, double-network (DN) architectures have been introduced. DN hydrogels combine rigid and flexible subnetworks to enhance network confinement while preserving mechanical properties. Jin et al. [60] constructed a PAMAA/CMC-Na/Fe DN hydrogel that showed minimal swelling across a wide pH range (pH 2–11), with an elastic modulus of 1.6 MPa and toughness of 0.24 MJ m^−3^. Xun et al. [38] further incorporated a zwitterionic network, where charge self-balancing reduced swelling ratios to ~4% and ~14.3% after 14 days in deionized water and high-salinity environments, respectively, while maintaining an elongation at break of 633%. Deng et al. [22] developed an SBMA/AA–PVA DN hydrogel (Figure 1B), in which enhanced hydrogen bonding lowered the swelling ratio from ~160% to ~80% while retaining a tensile strength of 0.76 MPa and elongation at break of 322%. Despite effectively balancing anti-swelling behavior and mechanical performance, DN hydrogels still rely on relatively simple crosslinking hierarchies, leaving considerable room to further improve long-term structural stability and environmental adaptability.

To improve network stability, increasing efforts have focused on constructing multi-crosslinked hydrogel systems integrating physical and chemical interactions. Chen et al. [61] developed a multiscale crosslinked network combining hydrogen bonding and ionic coordination (Figure 1C), enabling the hydrogel to retain ~96% of its mechanical properties at swelling equilibrium while reaching a tensile strength of 8.22 MPa. Compared with conventional DN hydrogels, such architectures generally offer higher structural integrity and stronger resistance to swelling-induced degradation. Beyond increasing crosslinking levels, introducing topological constraints has become an effective strategy to suppress swelling. Zhu et al. [62] designed a highly entangled double-network hydrogel, where chain entanglement generated additional topological confinement, allowing the material to maintain a tensile strength of ~3 MPa, a fracture energy of 8340 J m^−2^, and nearly complete mechanical reversibility at ~90% water content. To address the limited tunability of entangled systems, subsequent studies further enhanced intermolecular interactions through multicomponent synergistic design. Deng et al. [63] constructed an AA/SBMA/LMA copolymer network that achieved a swelling ratio of only 59.36% after 120 h of immersion. Recently, composite networks with ordered architectures have emerged as an important direction for anti-swelling hydrogel design. Huang et al. [64] incorporated covalent organic frameworks into an organic–inorganic hybrid network, reducing the swelling ratio to 0.4–2.5% after 10 days of immersion while retaining 92% of strength and 95.5% of toughness. Inspired by the hierarchical organization of biological tissues, biomimetic scaffold-reinforced networks have also been developed. Zhang et al. [65] proposed a plant-cell-wall-inspired “skeleton–filling” structure, where structural confinement and synergistic reinforcement reduced the swelling ratio to 0.385 without sacrificing ion transport efficiency. In addition to network reinforcement and scaffold-assisted strategies, interpenetrating architectures have shown strong potential for improving anti-swelling performance. Di et al. [8] fabricated an MXene-based composite hydrogel using a PAM/SA semi-interpenetrating polymer network (Figure 1D). Secondary crosslinking induced by the “SA–Ca^2+^–MXene” interaction reduced the equilibrium swelling ratio from 93.3% to 10.8%, and further to 2.56% under physiological saline conditions. Extending this concept, interpenetrating polymer networks (IPNs) further enhance topological confinement through mutual penetration of independently crosslinked networks. Shen et al. [11] reported an IPN hydrogel composed of β-CD-X-g-PAA, CMC, and PNIPAM, which maintained a swelling ratio below 5% after immersion in phosphate-buffered saline (PBS) at 37 °C for 72 h while exhibiting an elongation at break above 3600% and a compressive strength of 209 kPa.

### 2.2. Surface-Hydrophobic Structures

**Figure 2:**
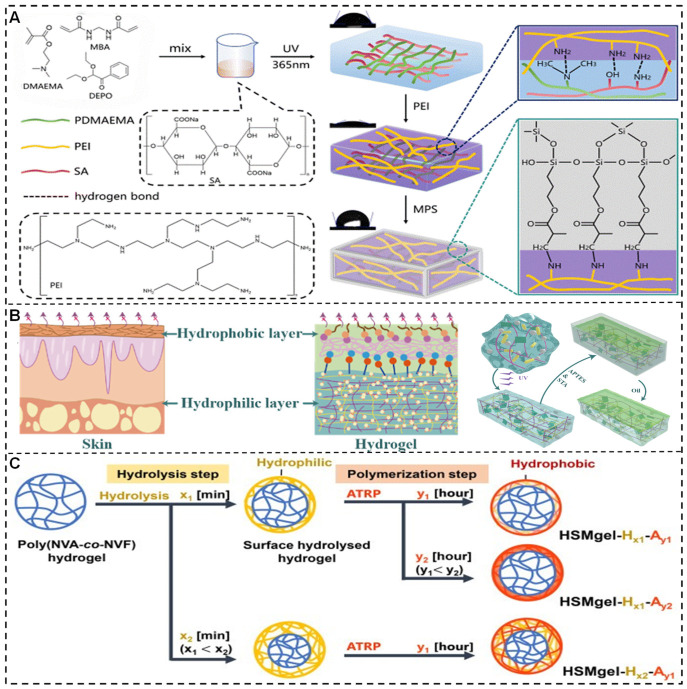
Design strategies of anti-swelling hydrogels through hydrophobic architecture engineering. (**A**) Hydrophobic surface layer design based on surface covalent modification [41]. (**B**) Hydrophobic surface layer design based on biomimetic skin-inspired architectures [42]. (**C**) Hydrophobic surface layer design based on surface-selective reactions [68].

Unlike bulk network confinement, surface-hydrophobic structures suppress hydrogel swelling through interfacial regulation by limiting water penetration. Introducing a hydrophobic barrier layer reduces surface wettability and delays water diffusion into the network, thereby mitigating both initial water uptake and long-term swelling [66]. This strategy is particularly effective in perspiration-rich, underwater, and humid environments, where hydrogel failure often originates from interfacial hydration, adhesion degradation, and signal instability. The most straightforward method is to construct a dense hydrophobic coating on the hydrogel surface. Yu et al. [41] fabricated a covalently bonded polysiloxane layer on a PDMAEMA/SA DN hydrogel. An interfacial transition layer was first formed using polyethyleneimine (PEI), followed by in situ generation of a Si–O–Si network via MPS conjugate addition and silane hydrolysis (Figure 2A). The resulting coating effectively inhibited water diffusion, decreasing the equilibrium swelling ratio from ~800% to ~40%, while restricting water loss in air to ~35% after 96 h. However, this strategy relies on multistep interfacial reactions and requires precise control over coating uniformity and thickness. To simplify interfacial engineering, interface-induced reconstruction strategies have been developed. Yi et al. [67] proposed a dynamic network reconstruction approach based on interfacial kinetics. Flexible hydrophobic siloxane chains grafted onto the mold surface induced polymer rearrangement during gelation, enabling in situ formation of a hydrophobic surface layer. Without changing the bulk composition, the hydrogel contact angle increased from ~35° to ~120°, achieving effective surface hydrophobization while maintaining bulk properties. Compared with conventional surface modification, this method reduces additional fabrication steps, although its effectiveness remains dependent on interfacial conditions and processing parameters.

Building upon interfacial hydrophobization, recent studies have shifted toward synergistically optimizing interfacial water resistance and internal functionality. Huang et al. [42] developed a biomimetic Janus bilayer hydrogel with an “outer hydrophobic–inner hydrophilic” structure (Figure 2B), where the hydrophobic outer layer blocked water penetration and ion exchange, while the inner hydrophilic conductive network maintained conductivity and interfacial adhesion. Through spatial decoupling of interfacial protection and bulk function, the hydrogel achieved an ultralow swelling ratio (~3.8%) and retained stable performance in aqueous environments, demonstrating the feasibility of simultaneously improving anti-swelling behavior and functional integrity. As interfacial design strategies continue to evolve, research has further advanced from homogeneous hydrophobic interfaces to spatially programmable architectures for more precise regulation of water transport. Yoshida et al. [68] fabricated a chemically bonded hydrophobic surface via in situ selective reaction and subsequently introduced poly(ethyl acrylate) through atom transfer radical polymerization, forming a gradient hydrophobic structure from the surface to the interior (Figure 2C). This design enhanced surface hydrophobicity while preserving the intrinsic hydrophilicity of the internal network, increasing the water contact angle from 17.6° to 91.9% and maintaining the swelling ratio at 22.8–28.6. Compared with conventional homogeneous hydrophobic coatings, gradient hydrophobic structures provide more refined control of interfacial water transport without sacrificing bulk properties, reflecting the evolution of hydrophobic interface design from simple water blocking to functional and precision-regulated interfacial engineering.

### 2.3. Core–Shell Structures

Core–shell structures suppress hydrogel swelling through spatial separation of functions between an external protective shell and an internal compliant network, thereby balancing water resistance with mechanical adaptability [69]. Different from conventional surface-hydrophobic coatings that primarily block water at the interface, core–shell architectures rely on synergistic regulation between the outer shell and the inner hydrogel matrix. Typically, the shell is designed with higher crosslinking density, lower water permeability, or stronger mechanical constraint to retard water penetration, while the inner network preserves flexibility, toughness, and conductivity to maintain deformation and sensing performance. In early work, Geng et al. [43] constructed a protective shell by introducing a stearic acid interfacial layer onto PVA/SA DN fibers followed by encapsulation with hydrophobic silicone rubber. This design reduced the swelling ratio to ~1.0% after 7 days of water immersion and effectively alleviated dehydration under ambient conditions. Nevertheless, the anti-swelling performance remained highly dependent on interfacial adhesion, and insufficient interfacial stability limited long-term durability. To enhance structural integration between the shell and the internal matrix, in situ fabrication strategies were subsequently developed. Li et al. [70] proposed a shell-powder-assisted in situ gelation method, where Ca^2+^-induced ionic crosslinking of sodium alginate enabled direct formation of a uniform hydrogel shell with a thickness of approximately 200–800 μm on the substrate surface. Compared with conventional coating approaches, in situ growth improved interfacial bonding and structural stability. However, such designs still mainly relied on overall encapsulation and provided limited regulation over the functionality of the internal network.

**Figure 3:**
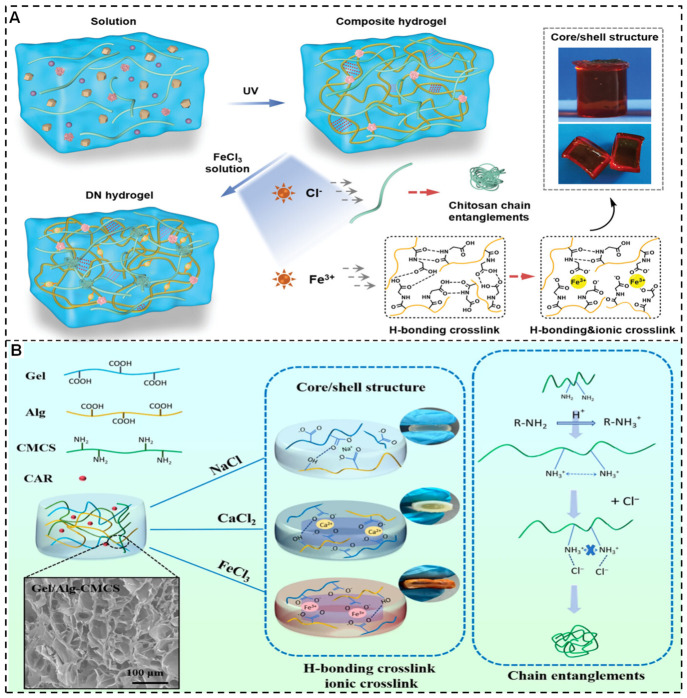
Design strategies of anti-swelling hydrogels through core–shell architecture engineering. (**A**) Design of a shell-structured CS/PACG DN anti-swelling hydrogel [44]. (**B**) Design of core–shell anti-swelling hydrogels regulated by multivalent ions [71].

To achieve synergistic regulation of swelling and stress distribution, core–shell hydrogels have evolved from simple surface encapsulation to spatially differentiated architectures. Dou et al. [44] fabricated a core–shell hydrogel via Fe^3+^-induced gradient diffusion (Figure 3A), forming a highly crosslinked coordination network and chain-entangled shell while retaining a relatively soft inner network. This heterogeneous structure reduced the equilibrium swelling ratio to 5.8–14.8% and maintained a compressive strength of 63.9–67.7 MPa after 30 days of immersion, demonstrating excellent dimensional stability and long-term durability. Based on this strategy, subsequent studies further explored controllable shell construction. Zhao et al. [71] introduced metal ions with different valence states (Na^+^, Ca^2+^, and Fe^3+^) and induced their diffusion from the surface inward to construct core–shell hydrogels (Figure 3B). The results showed that multivalent ions, especially Fe^3+^, produced a denser and more uniform highly crosslinked shell, whereas monovalent ions provided limited reinforcement. These findings suggest that coordination strength is a key factor governing anti-swelling performance and offer an effective strategy for precise regulation of core–shell structures. With further development of structural design, anti-swelling hydrogels have progressed from distinct shell–core separation to layered architectures with gradual transitions. Wang et al. [72] developed a hierarchical coating system through metal ion-induced polymerization and microphase separation, followed by the introduction of TA–Fe^3+^ coordination to construct a multilevel network. The obtained hydrogel exhibited long-term resistance to swelling and excellent cyclic stability in aqueous environments, while forming a continuous structural gradient from a dense outer layer to a relatively loose interior. Compared with conventional core–shell structures, this layered architecture alleviates abrupt interfacial changes and improves water transport regulation, representing a transition from binary compartmentalization to more refined structural control.

### 2.4. Gradient Structures

**Figure 4:**
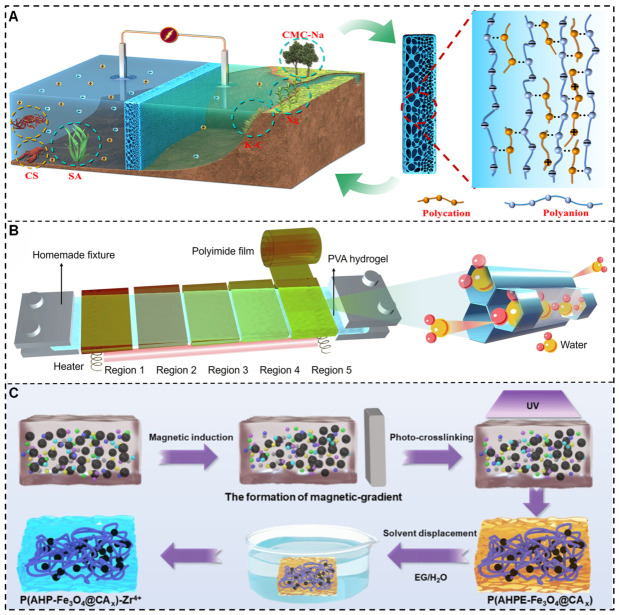
Design strategies of anti-swelling hydrogels through gradient architecture engineering. (**A**) Anti-swelling hydrogel design based on a continuous gradient architecture [47]. (**B**) Anti-swelling hydrogel design based on DAC gradient architecture [74]. (**C**) Anti-swelling hydrogel design based on magnetically responsive gradient architecture [46].

Gradient structures represent an advanced evolution of core–shell architectures, featuring continuous spatial variations in crosslinking density, pore morphology, charge distribution, mechanical properties, and hydrophilic–hydrophobic characteristics [73]. Compared with conventional layered or core–shell designs, gradient architectures eliminate abrupt interfacial transitions, reduce local stress concentration, and enable more homogeneous regulation of water transport. As a result, gradient engineering has become an effective strategy for enhancing the long-term dimensional stability of anti-swelling hydrogels. Initial gradient designs mainly relied on reaction–diffusion mechanisms to establish continuous charge distributions. Bian et al. [47] fabricated a charge-gradient hydrogel with progressively increasing charge density via interfacial reactions and molecular diffusion in a CS/SA/polysaccharide system (Figure 4A), forming a smooth transition from dense to porous regions across the thickness direction. Benefiting from the synergistic effects of charge gradients and ionic complexation, the hydrogel effectively regulated water migration and exhibited significant deswelling behavior under saline conditions. Notably, swelling was further suppressed with increasing ion concentration, demonstrating excellent resistance to swelling. Nevertheless, charge-gradient regulation mainly governs water transport through ion redistribution and offers limited control over the intrinsic network structure. Accordingly, gradient design has evolved toward structural and mechanical modulation. Zhu et al. [74] developed a mechanically graded PVA hydrogel with a continuous soft-to-hard transition using a directional annealing casting (DAC) strategy (Figure 4B). Controlled water migration induced polymer chain rearrangement and crystalline domain redistribution, generating a continuous network gradient from loose to dense structures within a single hydrogel. With increasing crystallinity and network compactness, the equilibrium swelling ratio decreased from ~60% to ~30% across different regions. More importantly, the integrated gradient hydrogel exhibited a total water uptake of only 5.39 g after 16 h, markedly lower than the theoretical cumulative value of individual regions (7.78 g), confirming that continuous structural gradients effectively suppress localized volumetric expansion and substantially improve overall dimensional stability.

Although structural gradients effectively regulate swelling behavior, their formation often depends on chain rearrangement and thermal treatment, limiting precise control and long-term stability. To address these challenges, external-field-assisted assembly combined with structural fixation has been developed. Xu et al. [46] aligned Fe_3_O_4_@CA nanoparticles under a magnetic field and immobilized the gradient architecture through Zr^4+^-mediated multipoint coordination (Figure 4C), constructing a stable magnetically responsive gradient hydrogel. The hydrogel achieved an ultralow swelling ratio of 2.7% and maintained a nanoparticle loss rate of only 1.27% after 15 days of immersion, demonstrating superior structural stability compared with homogeneous systems. Despite enabling precise gradient construction, external-field approaches require additional processing and post-fabrication stabilization. Therefore, recent studies have shifted toward spontaneous gradient formation without external intervention. Tian et al. [75] developed a self-organized gradient hydrogel using a self-floating polysiloxane crosslinker, which spontaneously redistributed network composition and crosslinking density along the thickness direction. This resulted in a gradual increase in pore size (2.74–14.80 μm) and porosity (3.78–32.14%), accompanied by a decrease in swelling ratio from 1083% to 442.9%. These findings indicate that spatial regulation of crosslinking density provides an additional structural dimension for controlling swelling behavior.

Overall, these four structural strategies achieve effective synergistic regulation of dimensional stability, mechanical integrity, and sensing stability, reflecting the evolution of anti-swelling design from uniform network reinforcement toward spatially organized functional regulation. Nevertheless, each strategy still has inherent limitations. Network-confinement structures often compromise flexibility and sensing sensitivity; surface-hydrophobic and core–shell structures rely heavily on interfacial integrity and are therefore susceptible to barrier failure or interfacial damage during long-term service; although gradient structures provide superior spatial regulation, their fabrication complexity and scalability remain major challenges. Therefore, future development of structurally engineered anti-swelling hydrogels should shift from static swelling suppression toward the regulation of dynamic service stability, achieving a higher level of synergistic optimization among water transport regulation, mechanical flexibility, conductive network retention, and interfacial stability.

## 3. Material Regulation Mechanisms for Anti-Swelling Hydrogels

Structural engineering strategies for anti-swelling hydrogels primarily include network confinement, surface hydrophobicity, core–shell architectures, and gradient structures. These strategies enhance the dimensional stability and environmental adaptability of hydrogels by optimizing network architecture, water transport pathways, and stress distribution. However, in the practical design of anti-swelling hydrogel sensors, structural engineering alone is often insufficient to simultaneously ensure long-term electrical stability, robust interfacial adhesion, and sustained sensing performance under complex hydrated environments. Therefore, structural engineering is typically integrated with material regulation strategies. Building upon a stable network architecture, material regulation further optimizes the internal water environment, reduces the driving force for water exchange, and preserves the continuity of conductive pathways and long-term interfacial stability through mechanisms such as ionic/coordination crosslinking [76,77], nanoconfinement [23], zwitterionic hydration [78,79], and solvation-mediated anti-water exchange [80,81,82]. Despite their common objective of suppressing uncontrolled water exchange, these material regulation strategies differ substantially in their molecular interactions, regulatory mechanisms, and resulting effects on network stability, conductivity retention, interfacial stability, and multifunctional integration (see Table 2).

### 3.1. Ionic/Coordination Crosslinking–Mediated Densification Mechanism

**Figure 5:**
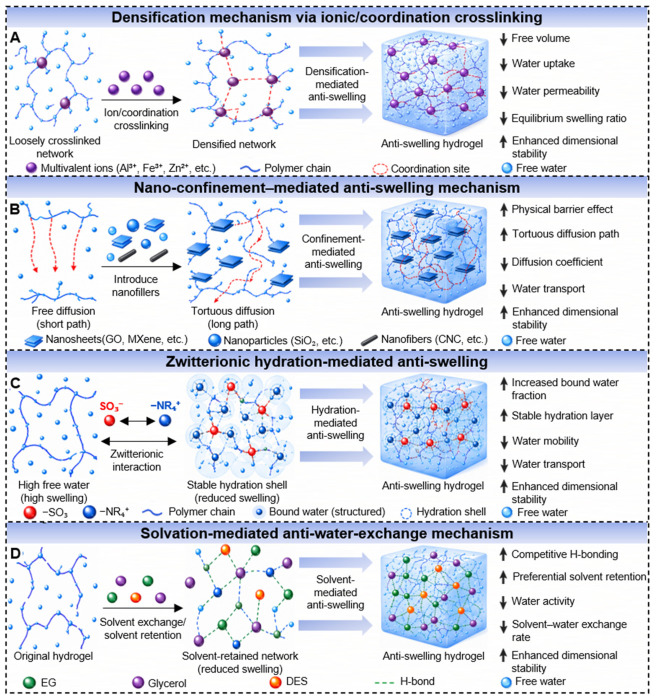
Representative regulation mechanisms of anti-swelling hydrogels. (**A**) Ion-/coordination-induced network densification [94,95,96]. (**B**) Nanoconfinement-mediated anti-swelling mechanism [23,41]. (**C**) Zwitterionic hydration-mediated anti-swelling mechanism [28,111,112]. (**D**) Solvation-mediated suppression of solvent–water exchange [81].

Ionic and coordination crosslinking is among the most effective and widely used strategies for constructing anti-swelling hydrogels. This approach relies on multipoint ionic or coordination interactions between multivalent ions and polymer functional groups (e.g., carboxyl, hydroxyl, and amino groups), which increase effective crosslinking density, reduce network free volume, and constrain polymer chain relaxation. Consequently, osmotic pressure-driven water uptake is intrinsically suppressed. Network densification further limits water penetration and diffusion, decreases the proportion of free water, and results in reduced swelling kinetics and lower equilibrium swelling ratios, thereby improving dimensional stability and long-term structural integrity (Figure 5A) [94,95,96]. Multivalent metal ions generally exhibit stronger anti-swelling capability due to their higher coordination capacity, while differences in ionic valence and coordination strength lead to distinct network regulation effects. For instance, Hu et al. [97] incorporated Al(OH)_3_ nanoparticles into a PAA/PSBMA copolymer network to construct a reversible physically crosslinked network through dynamic coordination bonds and hydrogen bonding, thereby achieving a synergistic integration of structural engineering and material regulation. The PAA/PSBMA network effectively regulated volume variation under saline conditions by balancing the polyelectrolyte and antipolyelectrolyte effects, while the Al(OH)_3_ nanoparticles further formed dynamic coordination bonds with carboxyl groups, enhancing network stability and suppressing salt-ion penetration. In artificial seawater, the hydrogel exhibited an equilibrium swelling ratio (V_2_/V_1_) of only 1.32, demonstrating excellent dimensional stability, while maintaining a tensile strength of approximately 1.2 MPa and an elongation at break of approximately 1100%. Similarly, Feng et al. [83] induced the salting-out contraction of cellulose chains using Al_2_(SO_4_)_3_ and subsequently stabilized the collapsed structure through Al–O–C coordination, enabling the hydrogel to maintain equilibrium swelling ratios of only 12.31% and 11.85% after 25 d of immersion in deionized water and saline solution, respectively. The hydrogel also exhibited consistently low swelling over a wide pH range (2–14), with an absolute swelling ratio (|SR|) below 14%, while retaining stable compressive mechanical properties after prolonged immersion. Ag nanowires were further incorporated into the hydrogel to fabricate a flexible strain sensor. The dense and stable anti-swelling network effectively preserved the continuity of conductive pathways, enabling the sensor to maintain stable responses over a strain range of 0–65% (GF = 0.34 and 0.71). Moreover, the sensor exhibited excellent signal reproducibility after 130 loading–unloading cycles and was successfully applied to human joint motion monitoring. These results demonstrate that anti-swelling not only suppresses volumetric expansion but also enhances conductivity stability, cyclic durability, and long-term signal reliability by mitigating water-induced network reconstruction and disruption of conductive pathways. Nevertheless, although a higher ionic coordination density is beneficial for further suppressing water uptake and reinforcing network stability, it may also restrict polymer chain mobility, reduce network flexibility, and increase the risk of embrittlement under hydrated conditions.

Compared with Al^3+^-based systems that suppress swelling mainly through network contraction, Fe^3+^ regulation relies more on dynamic multipoint coordination and cooperative network densification. Wang et al. [98] introduced TA–Fe^3+^ coordination into a poly(AM-co-MPC)/cellulose DN hydrogel, where metal–polyphenol interactions combined with rigid framework confinement effectively limited water transport. Consequently, the 7-day swelling ratio decreased from 735.9% to 50 ± 20% (≈93.2% reduction), while retaining a mechanical strength of 71.7 kPa and conductivity of 0.17 S m^−1^. Likewise, Zhao et al. [84] coupled Fe^3+^ coordination with polymer chain entanglement to further compact the network, converting positive swelling into negative swelling behavior (SR ≈ −10% to −12%) and maintaining nearly constant volume during prolonged immersion in seawater and physiological media. Beyond increasing effective crosslink density, Fe^3+^ also stabilizes the network dynamically to sustain anti-swelling performance. Chen et al. [99] combined Fe^3+^–carboxyl coordination with steric confinement to construct a low-modulus hydrogel, reducing the equilibrium swelling ratio to ~7% while preserving >90% of the initial mechanical properties after 24 h immersion. These results demonstrate that dynamic coordination can effectively suppress swelling without sacrificing structural compliance. With increasing ionic valence, hydrogel regulation shifts from reversible coordination toward stronger structural fixation. Although such evolution further suppresses water uptake, excessive constraint may limit network rearrangement and long-term stability. Zr^4+^-based systems exemplify this behavior. Wei et al. [85] constructed a highly compact network through Zr^4+^ coordination with carboxyl groups and oxygen-containing sites on MXene nanosheets, together with two-dimensional confinement, achieving an ultralow cumulative swelling ratio of 1.74% after 30 days and maintaining the normalized swelling ratio close to unity. Wang et al. [100] further strengthened a PAA/SA/GT/ carbon nanotube composite hydrogel via Zr^4+^ coordination, reducing the 14-day swelling ratio from ~180% to ~1%, accompanied by progressive pore shrinkage from 25.45 μm to 10.46 μm and finally to 5.63 μm. These findings indicate that high-density coordination and sustained pore compression can drive hydrogels toward a near non-swelling state. By contrast, divalent ions such as Zn^2+^, Cu^2+^, and Ca^2+^ possess weaker coordination strength but still inhibit swelling through dynamic crosslink exchange and network reconstruction. Zn^2+^ coordination reduced the 72 h swelling ratio from ~370% to ~200% [101]; freeze-induced Cu^2+^–SA coordination reorganization lowered equilibrium swelling from ~240% to 32.26% [102]; and Ca^2+^-mediated ionic/electrostatic dual crosslinking decreased the swelling ratio from ~3.2 to ~1.7 while restricting final volume expansion to ~1.3-fold [103]. Ionic/coordination crosslinking constructs dense polymer networks through multivalent coordination interactions and has emerged as one of the most effective material-based strategies for enhancing the anti-swelling performance of hydrogels. Nevertheless, an inherent trade-off remains between anti-swelling capability and network dynamics. While multivalent ions can establish more stable coordination networks that effectively suppress swelling, they may also compromise the dynamic restructuring of the network. In contrast, low-valence ions provide greater dynamic reversibility but are less capable of maintaining a highly compact network over prolonged operation. Future efforts should therefore focus on developing coordination systems that simultaneously integrate structural stability and dynamic adaptability, thereby enabling the synergistic enhancement of long-term anti-swelling performance and functional stability.

### 3.2. Nanoconfinement-Mediated Anti-Swelling Mechanism

The anti-swelling behavior mediated by nanofillers primarily originates from nanoscale spatial confinement, interfacial interactions, and regulation of water transport. Unlike conventional crosslinking strategies that suppress swelling through network densification, nanoconfinement regulates swelling by reconstructing mass transport pathways. Specifically, nanofillers create physical barriers and confined diffusion domains that prolong water migration distance, increase transport tortuosity, and reduce the effective diffusion coefficient, thereby suppressing continuous water uptake and limiting volumetric expansion (Figure 5B) [23,41]. Such transport-dominated regulation enables long-term dimensional and functional stability while largely preserving hydrogel hydration and mechanical compliance. Representative fillers—including two-dimensional nanosheets, nanoparticles, nanofibers, and carbon-based materials—can be incorporated into hydrogel networks to form localized confinement regions and diffusion barriers. These architectures reduce pore interconnectivity, restrict polymer chain relaxation, and modulate water transport behavior, ultimately enhancing swelling resistance. Among them, two-dimensional nanosheets exhibit particularly strong confinement capability owing to their high aspect ratio and long-range diffusion resistance. Zhu et al. [104] developed a low-swelling adhesive GelMA–Laponite–TA hydrogel (GNT hydrogel), in which Laponite nanosheets cooperated with TA-mediated hydrogen bonding to compress the pore structure and impede water penetration. Under artificial saliva conditions, pristine GelMA exhibited a swelling ratio of 1186.9%, whereas GNT hydrogel reduced swelling to approximately one-eighth of the original value and retained more than 88% of its mass after 3 days of immersion. Similarly, Xuan et al. [105] introduced Laponite to construct a “house-of-cards” confinement architecture, reducing the swelling ratio from ~280% to ~115% (≈58.9% reduction), while simultaneously increasing tensile strength from 76 to 270 kPa and compressive strength from 81 to 161 kPa. These findings demonstrate that nanosheet-mediated confinement can simultaneously suppress swelling and reinforce mechanical performance. Building upon this strategy, Yang et al. [27] further constructed a nanoconfinement network through the synergistic incorporation of Laponite nanosheets and graphene oxide (GO) nanosheets, effectively enhancing the stability of the polymer network. Combined with subsequent LiCl substitution to further suppress water uptake, the equilibrium swelling ratio of the hydrogel was reduced from 315% to 157%, ultimately achieving negative swelling (−41%). On this basis, the resulting sensor exhibited a high electrical conductivity of 4.3 S·m^−1^, a maximum GF of 3.04, and stable electrical output during cyclic stretching. Notably, swelling-induced polymer chain expansion typically leads to the reconstruction of conductive networks, resulting in the disruption of conductive pathways and resistance drift. In contrast, the nanoconfinement network constructed by Laponite and GO effectively suppressed this structural evolution, thereby preserving the continuity of conductive pathways and providing structural support for stable electrical conductivity and strain responses. Nevertheless, excessive nanosheet loading may reduce water retention, induce network stiffening, and delay interfacial response, thereby compromising conformability.

Beyond nanosheets, nanoparticles mainly generate localized barriers by occupying network free volume and reducing pore connectivity. Mohammed et al. [106] incorporated vinyl-functionalized SiO_2_ nanoparticles into a PAMPS/PAAm DN hydrogel, decreasing water content from 96.73% to 87.89% and reducing the swelling ratio from 2757% to 1220% (≈55.7% reduction). Likewise, Newham et al. [107] reinforced hyaluronic acid hydrogels using PEI-functionalized silica nanoparticles, where particle filling and electrostatic interactions promoted the formation of thicker pore walls and denser architectures. Although the reduction in swelling was relatively limited, improved structural integrity and increased network density suggested that localized occupancy effectively delayed volumetric expansion. However, in the absence of continuous barrier formation, nanoparticle-based systems generally exhibit weaker long-term regulation of water transport than high-aspect-ratio nanosheets. Compared with discrete nanoparticles, two-dimensional carbon materials provide stronger long-range diffusion resistance. Zainab et al. [108] introduced GO into a silane-crosslinked chitosan/sodium alginate hydrogel, where GO nanosheets and the Si–O–Si confinement framework synergistically reduced pore connectivity. The hydrogel exhibited a maximum swelling ratio of approximately 100% at pH 6, which further decreased with increasing ionic strength and reached ~0.9 in CaCl_2_ solution, while maintaining a gel fraction of 89%. These results indicate that carbon-based barriers can effectively preserve network integrity under aqueous environments. Further enhancement of swelling resistance can be achieved through nanofiber and rigid scaffold systems that emphasize continuous spatial confinement. Fu et al. [109] developed a lignocellulose nanofiber-reinforced deep eutectic solvent (DES) hydrogel, where fiber entanglement and hydrophobic barriers cooperatively suppressed water migration. The optimized LCNF5–gel exhibited an equilibrium swelling ratio of only ~25% after 12 days in deionized water, substantially lower than that of the pristine DES system (~2252–2300%), while retaining a tensile strength of ~1.13 MPa compared with only ~0.012 MPa for the control. Similarly, Lan et al. [86] employed a highly crystalline cellulose framework (CrI = 87.3%) with compact pores (0.1–35 μm), reducing equilibrium swelling ratios to 38% and 48% in seawater and deionized water, respectively. Nyamayaro et al. [110] further established a continuous confinement network using cellulose nanocrystals, lowering equilibrium swelling from ~42 to ~30 g g^−1^ (≈28.6% reduction), with further enhancement achieved through multivalent ion reinforcement. Unlike ionic crosslinking, which directly reinforces the polymer network, nanoconfinement-mediated anti-swelling primarily relies on nanofillers to regulate water transport pathways. Consequently, its effectiveness is largely governed by the continuity and integrity of the confinement architecture. Two-dimensional nanosheets establish long-range diffusion barriers that effectively prolong water migration pathways, whereas nanoparticles mainly reduce local pore interconnectivity. In contrast, nanofibers provide continuous spatial confinement throughout the hydrogel network. Although each type of nanofiller exhibits distinct advantages, balancing homogeneous dispersion, interfacial compatibility, and the preservation of mechanical flexibility under high filler loadings remains a major challenge for advancing nanoconfinement-mediated anti-swelling strategies.

### 3.3. Zwitterionic Hydration-Mediated Anti-Swelling

The anti-swelling behavior of zwitterionic hydrogels primarily originates from their unique hydration regulation capability. Zwitterionic polymer chains simultaneously contain positively and negatively charged groups that generate stable hydration shells through electrostatic coupling and thereby regulate the balance between free and bound water. Unlike strategies that suppress swelling through network densification or diffusion confinement, zwitterionic systems maintain dimensional stability by increasing the proportion of bound water while reducing the content of mobile free water. Such water-state regulation enables the coexistence of high hydration and low swelling, ultimately improving long-term structural stability and environmental adaptability (Figure 5C) [28,111,112]. For example, Zheng et al. [28] developed a PVA/PSBMA double-network anti-swelling hydrogel. The double-network architecture effectively suppressed water diffusion by increasing the crosslinking density, restricting polymer chain mobility, and forming a stable three-dimensional framework. Meanwhile, the zwitterionic nature of PSBMA optimized the internal network interactions through electrostatic interactions and hydrogen bonding, while phytic acid further enhanced the noncovalent interactions. After immersion in artificial seawater for 14 d, the hydrogel exhibited an equilibrium swelling ratio of only 1.8%, while maintaining a tensile strength of 304 kPa, an elongation at break of approximately 830%, and an ionic conductivity of 5.8 mS·cm^−1^. In addition, the hydrogel maintained stable electrical responses after 900 stretching cycles. These results indicate that zwitterionic hydration regulation can effectively suppress long-term swelling without sacrificing water retention. Beyond bound-water regulation, zwitterionic systems often exhibit superior dimensional stability under high-salinity environments. Zhang et al. [113] constructed an ionically conductive anti-swelling P(SBMA-co-AA)/Al^3+^ hydrogel, where SBMA established a continuous hydration network and elevated ionic strength further enhanced internal charge shielding to suppress chain expansion. Consequently, equilibrium swelling ratios decreased to ~0.69 in artificial sweat and ~0.57 in seawater, accompanied by a reduction in water contact angle from 61° to 15.3%. These findings suggest that strengthening hydration interactions can significantly improve long-term environmental stability under complex aqueous conditions. Further evidence for hydration-mediated regulation was reported by Duan et al. [90], who developed a gel/SBMA/AAm/AAc/AlCl_3_ conductive hydrogel. In this system, SBMA promoted the conversion of free water into bound water while simultaneously strengthening interchain interactions and network stability, enabling persistent low-swelling behavior during prolonged immersion. Notably, the optimized hydrogel retained an elongation at break of ~1053.8%, tensile strength of 252.7 kPa, and ionic conductivity of 6.38 mS cm^−1^, demonstrating that hydration regulation can be achieved without substantial loss of mechanical or conductive performance.

However, increasing zwitterionic content does not necessarily lead to continuous improvement. Excessive hydration may further suppress swelling but can simultaneously reduce ion transport efficiency, weaken network modulus, and induce wet-state softening. Therefore, effective anti-swelling design requires balancing hydration stabilization, conductive transport, and mechanical support. To overcome structural softening caused by hydration enhancement alone, recent studies increasingly integrate zwitterionic regulation with reversible physical networks or dynamic crosslinking strategies. Such approaches preserve bound-water environments while introducing additional structural support to synergistically improve anti-swelling performance and long-term stability. Sun et al. [89] constructed a nanocomposite conductive hydrogel by incorporating the zwitterionic polymer PSBMA with carbon nanotubes (CNTs). Stable electrostatic interactions among the PSBMA chains formed a physically crosslinked network, while CNTs established continuous conductive pathways. The zwitterionic network effectively mitigated the disturbance of the polymer network induced by the aqueous environment, providing a stable conductive microenvironment for the CNTs and reducing the risk of conductive pathway reconstruction during swelling, thereby maintaining stable electrical responses under both wet and underwater conditions. Based on this design, the hydrogel maintained a swelling ratio of approximately 50% after 150 h of water immersion, which was further reduced to approximately 30% after the incorporation of CNTs. Meanwhile, the hydrogel achieved a maximum GF of 10.35, a pressure sensitivity of 0.256 kPa^−1^, and stable underwater strain-sensing performance. Similarly, Fu et al. [114] combined zwitterionic hydration regulation with Fe^3+^ dynamic crosslinking to simultaneously optimize internal water distribution and structural stability, reducing the minimum swelling ratio to ~33% and achieving excellent dimensional retention. Unlike strategies that enhance anti-swelling performance through network densification, the anti-swelling behavior of zwitterionic systems is governed by stable hydration shells that rebalance free and bound water. However, excessive hydration, while beneficial for suppressing swelling, may compromise interfacial adhesion and weaken intermolecular interactions. Therefore, precise regulation of hydration-shell thickness and ionic distribution remains essential for simultaneously optimizing anti-swelling performance, interfacial stability, and electrical conductivity.

### 3.4. Solvation-Mediated Anti-Water-Exchange Mechanism

Solvation regulation has emerged as an important strategy for constructing anti-swelling hydrogels in recent years. Its central concept is to reconstruct the internal solvent environment to reduce water activity and weaken the thermodynamic driving force for water exchange across the hydrogel interface [81,82]. Polyols [115,116], ionic liquids [117,118], DES-based eutectogels [119,120], and organic/water mixed solvents can regulate polymer–water interactions through competitive hydrogen bonding, preferential solvation, and hydrophobic solvent environments, thereby suppressing continuous ingress of external water into the network (Figure 5D) [81]. Unlike network densification or diffusion confinement, solvation-mediated regulation controls swelling at the solvent level and enables long-term dimensional and functional stability without excessively sacrificing flexibility or water retention. Among these approaches, low-molecular-weight polyols represent the most typical anti-water-exchange regulators. Chen et al. [91] developed a PVA/gelatin double-network anti-swelling hydrogel and introduced glycerol to regulate the internal solvent environment. The double-network architecture constructed a continuous and stable network framework, enhancing the cooperative constraint among polymer chains and effectively retarding water diffusion into the network. Meanwhile, glycerol formed stable hydrogen bonds with water molecules and partially replaced the free water within the hydrogel, thereby reducing water activity, suppressing internal and external water exchange, and further improving network stability. The hydrogel exhibited swelling ratios below 17% after 24 h of immersion in deionized water, 0.9% NaCl solution, and 0.5 M HCl. The stable internal solvent environment effectively mitigated water absorption-induced network reconstruction, providing reliable support for the MXene conductive network. Consequently, the hydrogel simultaneously exhibited an electrical conductivity of 1.4 S·m^−1^, a GF of 3.15, and stable performance in underwater human motion monitoring and bioelectrical signal acquisition. Similarly, Cerna et al. [121] increased glycerol content to promote PVA–glycerol interactions and induce the formation of a compact nonporous structure, reducing swelling from ~5.5 to ~2.0–2.4 (>50% reduction) while retaining ~50% structural integrity after 28 days at 37 °C. However, increasing polyol content does not necessarily lead to superior performance. Although stronger solvent–polymer interactions suppress water uptake, excessive solvent fixation may hinder ion transport and weaken environmental responsiveness. Therefore, solvent replacement alone is often insufficient to simultaneously achieve long-term stability and functional output. Ethylene glycol (EG)-based solvent exchange further strengthens anti-water-exchange capability. Guan et al. [122] developed an environmentally tolerant P(AMPS/HEMA/EG) anti-swelling hydrogel, in which solvent exchange transformed solvent-bridged hydrogen bonding into stronger interchain hydrogen interactions while reducing water-entry driving forces through hydrophilic–hydrophobic balance. Consequently, the swelling ratio remained as low as ~0.99% after 30 days in deionized water and further decreased to ~0.44% in 0.2 M NaCl solution, accompanied by a volume contraction of −44.05% at 60 °C. Likewise, Gao et al. [123] combined EG/water mixed solvents with lignin-induced hydrophobic barriers to regulate the solvation environment, reducing equilibrium swelling from ~23–23.7% to ~10%, while retaining elongation at break above 300%, tensile strength of 0.13 MPa, and conductivity of ~0.18 S m^−1^ after prolonged immersion.

Compared with conventional small-molecule solvent systems, DES-based eutectogels provide more systematic regulation of internal water environments. Zhang et al. [93] introduced a 1-butyl-3-methylimidazolium chloride–solketal DES into a P(SBMA-co-AA) network, where dynamic hydrogen bonding and competitive solvation restricted water migration and hydrophobic microdomains suppressed network expansion. The hydrogel maintained an equilibrium swelling ratio of only ~3% after 30 days in water and ~20% in artificial seawater, while preserving structural stability across pH 1–11 and retaining an elongation at break of ~1468% after prolonged immersion. Further evidence was provided by Wei et al. [124], who developed a choline chloride/EG-based DES eutectogels. In this system, DES preferentially formed strong hydrogen bonds with water and disrupted continuous water networks. Molecular simulations showed that the number of H_2_O–DES hydrogen bonds reached 709, exceeding the H_2_O–H_2_O network (485), with binding energy increasing to −9.57 kcal mol^−1^. Consequently, the swelling ratio remained below 2% after 7 days at pH 7, while flexibility and structural stability were preserved even at −60 °C. As solvation strength continues to increase, swelling can be further reduced but often at the expense of mass transport and interfacial adaptability. Therefore, recent research is gradually shifting from maximizing solvent immobilization toward constructing selective water-exchange environments. In this context, hydrophobic DES systems offer an alternative pathway by directly suppressing external water exchange at the solvent level. Zhang et al. [92] developed a hydrophobic DES-based anti-swelling eutectogel in which a continuous hydrophobic solvent phase and low-surface-energy barrier jointly inhibited water ingress. The eutectogel maintained water contact angles above 110°, while swelling ratios after 72 h immersion were only ~5% and ~2.6% for L/G–50% and L/G–70%, respectively. Even after 10 days of immersion, ionic conductivity remained ~0.18 mS cm^−1^. Solvation regulation has evolved from simple solvent replacement toward the precise regulation of the internal solvent environment. Unlike conventional crosslinking strategies, it suppresses swelling while largely preserving the intrinsic functionality of hydrogels. Nevertheless, substantial differences remain among solvent systems in their effects on ion transport, mass-transfer behavior, and interfacial compatibility. Therefore, achieving a synergistic balance among selective water exchange, stable functional output, and favorable biocompatibility will be essential for the continued development of solvation-mediated anti-swelling hydrogels.

Overall, building upon structural engineering, material regulation further optimizes the internal water environment and intermolecular interactions within hydrogels, thereby synergistically regulating water transport, network stability, and functional retention. This enables the suppression of swelling while simultaneously maintaining conductive networks, mechanical performance, and interfacial stability, reflecting the evolution of anti-swelling design from single structural optimization toward the synergistic integration of structural engineering and material regulation. Nevertheless, current material regulation strategies still exhibit several limitations. Enhancing network stability is often accompanied by reduced flexibility and sensing performance, while some systems continue to face challenges related to filler dispersion, biocompatibility, long-term stability, and scalable fabrication. Therefore, future development of anti-swelling hydrogels should further strengthen the integration of network architecture and material regulation, achieving more systematic synergistic optimization among water transport regulation, mechanical adaptability, conductivity retention, and long-term service stability.

## 4. Applications in High-Humidity Perspiration and Aquatic Motion Environments

Anti-swelling hydrogels are evolving beyond low-water-uptake soft materials toward reliable sensing interfaces capable of operating under complex humid environments. For wearable sensors, performance degradation under wet conditions rarely originates from a single factor; rather, it results from the coupled effects of continuous water uptake, interfacial hydration, conductive component redistribution, dynamic mechanical deformation, and external fluid disturbances. These processes collectively disrupt structural integrity, interfacial coupling, and signal transmission, ultimately compromising long-term sensing reliability. Accordingly, this section focuses on two representative application scenarios—high-humidity perspiration environments and underwater conditions—to discuss how anti-swelling hydrogels enhance the long-term reliability of wearable sensors under complex aqueous exposure. Particular emphasis is placed on their ability to preserve structural stability, maintain robust interfacial contact, and sustain conductive continuity, thereby enabling stable and reliable signal acquisition during prolonged operation.

### 4.1. Physiological Monitoring Under High-Humidity Perspiration Environments

**Figure 6:**
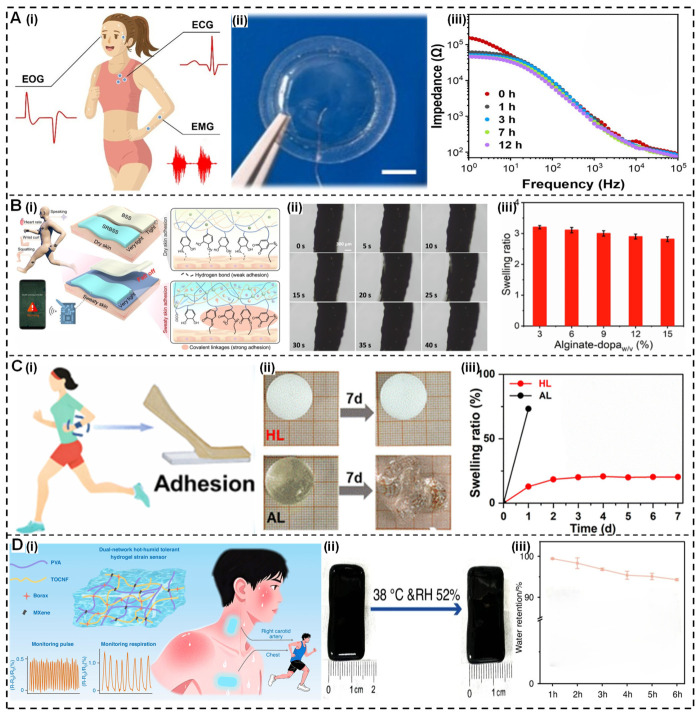
Applications of anti-swelling hydrogel sensors in high-humidity and perspiration environments. (**A**) Interface stability and sensing performance of BioSP-P hydrogel electrodes under high-humidity and perspiration conditions: (**i**) Schematic illustration of signal acquisition using BioSP-P hydrogel electrodes during motion; (**ii**) skin–electrode interfacial impedance measurements of BioSP-P hydrogel electrodes; (**iii**) time-dependent shear strength and adhesion retention of BioSP-P hydrogels [130]. (**B**) Interface stability and sensing performance of SRBSS hydrogel sensors in high-humidity and perspiration environments: (**i**) Schematic illustration of signal acquisition using SRBSS hydrogel sensors during motion; (**ii**) morphological comparison of HA and PHA organohydrogels before and after swelling for 15 d; (**iii**) swelling behavior of SRBSS hydrogels with different alginate–dopamine contents (3–15 *w*/*v*%) in PBS [131]. (**C**) Motion monitoring and sensing performance of HL hydrogels under high-humidity and perspiration conditions: (**i**) Interface adaptation and physiological monitoring of HL hydrogels in perspiration-rich environments; (**ii**) morphological evolution of anti-swelling HL hydrogels and conventional AL hydrogels after immersion for 7 d; (**iii**) performance comparison of HL and AL hydrogels before and after immersion [132]. (**D**) Real-time physiological monitoring and sensing performance of MXene DN hydrogels under high-humidity and perspiration conditions: (**i**) Interface stability and sensing performance of MXene hydrogel sensors under elevated temperature and humidity; (**ii**) performance evaluation of PBTM-3 at 38 °C and 52% relative humidity; (**iii**) stability changes of PBTM-3 after exposure to 38 °C and 52% relative humidity for 6 h [134].

During motion monitoring and long-term skin attachment, perspiration continuously alters the hydrogel–skin interfacial state and becomes a major source of sensing instability. Water and salt ions in sweat not only induce hydrogel swelling but may also generate interfacial hydration layers, leading to reduced adhesion strength, altered interfacial impedance, and baseline drift of physiological signals [125,126,127]. Therefore, anti-swelling design under high-humidity conditions should not be evaluated solely by maintaining low swelling ratios, but rather by its ability to preserve interfacial stability and reliable signal output under sweating, friction, and repetitive motion. To address sweat-induced interfacial failure, recent studies have introduced dynamic crosslinking and interfacial hydrophobic regulation to enhance wet-state adhesion. Zhang et al. [128] developed a PVA-Dopa/AM hydrogel in which catechol–borate dynamic crosslinking enabled perspiration-responsive adhesion regulation. Under simulated sweat conditions, the adhesion strength increased from 13.45 to 24.0 kPa (≈78% enhancement), while stable interfacial bonding was maintained during continuous motion and cyclic adhesion, enabling reliable human motion monitoring. Similarly, Shang et al. [129] constructed an AMPS/CS/TA/MEA conductive adhesive hydrogel, where a hydrophobic interfacial barrier reduced hydration-layer interference and maintained a wet adhesion strength of ~67 kPa together with ~73% mass retention after one week. Benefiting from this interfacial stability, the sensor achieved reliable monitoring of joint and laryngeal motion and preserved stable electrical responses over 400 loading cycles. Beyond interfacial regulation, improving bulk structural stability through network densification and water transport control has emerged as an effective strategy to maintain sensing performance under perspiration. Liang et al. [130] developed a sweat-enhanced DN electrode (BioSP-P) by integrating Ca^2+^-modified silk fibroin with a compact PAA-NHS network and introducing PDMS to improve water retention (Figure 6A(i–iii)). Under perspiration conditions, ionic conductivity increased from 1.01 to 1.46 S m^−1^, interfacial toughness improved from 411.66 to 504.64 J m^−2^, and shear strength reached 40.02 kPa, enabling continuous acquisition of high-quality electromyographic signals. Similarly, Li et al. [131] increased network crosslink density to construct the SRBSS hydrogel, which exhibited an ultralow swelling ratio of only ~2.7–3.2 after immersion in PBS and maintained stable attachment on dry, oily, and perspiring skin surfaces, allowing continuous electrocardiographic monitoring during running and jumping (Figure 6B(i–iii)). Moving beyond conventional anti-swelling regulation, Liu et al. [40] further proposed a non-swelling hydrogel (Gr–PMAm) with a swelling ratio consistently below 0.2 (compared with ~1.2 for conventional systems). Owing to its stable structure under humid conditions, the hydrogel enabled continuous monitoring of multiple physiological activities, including respiration, coughing, and joint movement.

Building on interfacial and bulk stability, structural engineering has recently been employed to optimize stress distribution and water transport behavior for prolonged sensing under humid environments. Zhang et al. [132] developed a bilayer that maintained structural integrity after 7 days of water immersion, whereas the control group exhibited severe swelling and structural failure. The equilibrium swelling ratio remained at only ~20%, significantly lower than that of conventional systems (~75%), enabling continuous motion monitoring with stable signal output under humid conditions (Figure 6C(i–iii)). Likewise, Liu et al. [133] constructed a DNA-inspired hydrogel based on A–U nucleobase pairing, which exhibited nearly unchanged volume after ~250 h exposure to humid environments while maintaining a shear adhesion strength of 5–8 kPa, allowing stable monitoring of finger, joint, and laryngeal movements. In addition to structural stability, suppression of signal drift during long-term humid exposure remains critical for reliable sensing. Wang et al. [134] developed a PVA/TOCNF/MXene/Borax conductive DN hydrogel, in which hydrogen bonding and dynamic borate interactions synergistically stabilized the conductive network. Under simulated exercise conditions (38 °C, 52% relative humidity (RH)), the sensor maintained stable resistance response and strain sensitivity (GF ≈ 7.8) after 6 h of continuous operation. Benefiting from its environmental stability, the device enabled continuous monitoring of pulse and respiration signals and accurately distinguished cardiopulmonary responses under different exercise intensities (heart rate: 82–125 bpm; respiratory rate: 22–39 rpm) (Figure 6D(i–iii)). Overall, although existing anti-swelling strategies have demonstrated improvements in interfacial stability and sensing performance under high-humidity perspiration environments, direct comparisons among different systems remain challenging because of inconsistencies in evaluation criteria and testing protocols. Therefore, a standardized evaluation framework for high-humidity perspiration environments is proposed to enable objective comparisons across different anti-swelling hydrogel systems. Within this framework, artificial sweat serves as the standard testing medium, with PBS employed as a complementary control to simulate perspiration and physiological environments, respectively. The testing conditions are standardized at 37 °C and 90 ± 5% RH, with evaluation periods of 1 h, 24 h, 7 d, and 30 d to assess both short-term responsiveness and long-term stability. In addition to the equilibrium swelling ratio, key evaluation metrics include mass retention, wet adhesion strength, interfacial impedance variation, conductivity retention, and continuous signal stability. Furthermore, comprehensive assessments should be conducted under representative dynamic exercise conditions, including running, repetitive joint bending, continuous perspiration, and skin friction, thereby systematically evaluating the structural stability, interfacial stability, and reliability of physiological signal monitoring exhibited by anti-swelling hydrogels under high-humidity perspiration environment.

### 4.2. Physiological Monitoring in Underwater Environments

**Figure 7:**
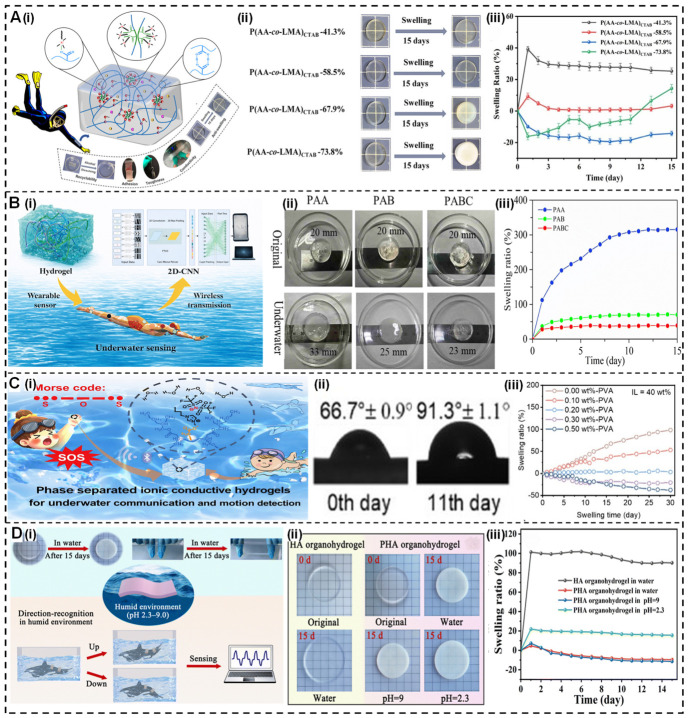
Applications of anti-swelling hydrogel sensors in underwater environments. (**A**) Stability and sensing performance of supramolecular anti-swelling hydrogels in complex aqueous environments: (**i**) Underwater sensing applications of supramolecular anti-swelling hydrogels; (**ii**) morphological evolution of different P(AA-co-LMA)/CTAB hydrogels after immersion in deionized water at 25 °C for 15 d; (**iii**) swelling behavior of different P(AA-co-LMA)/CTAB hydrogels [138]. (**B**) Stability and motion sensing performance of PABC DN conductive hydrogels in underwater environments: (**i**) Underwater wireless motion sensing based on PABC DN conductive hydrogels; (**ii**) morphological comparison of PAA, PAB, and PABC hydrogels after immersion for 15 d; (**iii**) variation in swelling ratio of different hydrogels during immersion [87]. (**C**) Motion monitoring and sensing performance of IL–PS hydrogels under underwater conditions: (**i**) Underwater communication and motion monitoring enabled by IL–PS hydrogels; (**ii**) evolution of water contact angle of pre-organogels during solvent exchange; (**iii**) swelling behavior of IL–PS hydrogels with different PVA contents [142]. (**D**) Stability and motion sensing performance of PVP/P(HEA-AA) organohydrogels in aqueous environments: (**i**) Underwater motion sensing applications of PHA organohydrogels; (**ii**) morphological evolution of HA and PHA hydrogels after immersion in water, acidic (pH 2.3), and alkaline (pH 9) environments for 15 d; (**iii**) swelling behavior of PHA hydrogels under different environmental conditions [143].

Compared with high-humidity perspiration environments, underwater conditions impose more stringent requirements on hydrogel-based sensors. During prolonged underwater exposure, hydrogels are continuously subjected to immersion, osmotic gradients, fluid erosion, and external mechanical disturbances, which increase the likelihood of volumetric swelling, conductive component loss, interfacial delamination, and signal attenuation [135,136,137]. Therefore, underwater sensing requires not only low-swelling behavior but also the ability to maintain mechanical integrity, conductive continuity, and stable deformation response after long-term immersion. Constructing compact and stable networks represents one of the most fundamental strategies for achieving underwater anti-swelling performance. Ji et al. [21] developed a mussel-inspired PSBMA/TA@MMT hydrogel in which zwitterionic interactions, nanoclay crosslinking, and multiple noncovalent interactions synergistically reinforced the network structure. The hydrogel exhibited a swelling ratio of only ~3% while maintaining an underwater adhesion strength of 15.10 kPa, enabling stable motion monitoring and wireless signal transmission. Similarly, Qi et al. [138] constructed a supramolecular P(AA-co-LMA)/CTAB network that maintained nearly unchanged volume after 15 days of immersion (swelling ratio ≈ 0%) despite a water content of 58.5%, while preserving low-swelling behavior in seawater, PBS, and pH 3–9 environments. Owing to its structural stability, the sensor maintained stable signal output across 0–100% strain without noticeable frequency attenuation (Figure 7A(i–iii)). Beyond structural densification, charge regulation has been introduced to suppress continuous water uptake by reducing osmotic imbalance between the hydrogel interior and external environments. Ren et al. [39] developed a PVA/P(SBMA-co-HEMA) DN hydrogel with an equilibrium swelling ratio of only ~9% after 30 days of immersion, while simultaneously achieving a toughness of 518 kJ m^−3^ and ionic conductivity of 4.58 S m^−1^. The resulting device enabled stable monitoring of head lifting, arm swinging, and joint bending. Likewise, Yu et al. [139] constructed a cation-regulated hydrogel network that reduced swelling to 15.3% and further induced volumetric contraction of −8.5% in seawater, enabling reliable underwater motion sensing and information transmission. As swelling resistance improves, long-term stability of conductive pathways becomes increasingly critical for sensing reliability. Bi et al. [140] developed a CS/ionic liquid DN hydrogel that maintained a negative swelling ratio of −3.79% even after 100 days of immersion, while ionic liquid-mediated conductive channels ensured persistent underwater monitoring and warning capability. Similarly, Huang et al. [26] employed metal coordination to construct a near-zero-swelling hydrogel (swelling ratio ≈ 0%), retaining approximately 88% of its toughness after 20 days underwater and supporting continuous signal acquisition and wireless communication.

Once structural integrity and conductive continuity are established, anti-swelling hydrogels can further support advanced information processing functions. Qi et al. [87] developed a conductive P(AA-co-SBMA)/BC DN hydrogel with an equilibrium swelling ratio of only ~11% after 15 days in deionized water, substantially lower than conventional systems (PAA: ~300%; PAB: ~50–70%). Based on its stable conductive network, the system achieved underwater motion sensing and wireless transmission and further integrated two-dimensional convolutional neural network analysis to classify 16 swimming postures with an accuracy of 99.37% (Figure 7B(i–iii)). More recently, anti-swelling strategies have begun integrating energy management and information interaction capabilities. Xiong et al. [141] introduced polyvinylidene fluoride to construct a self-powered hydrogel system with a swelling ratio of ~21.39%, enabling underwater signal acquisition and transmission without external power supply. Han et al. [142] further developed a bicontinuous conductive network through ionic liquid-mediated phase separation, maintaining conductivity of ~0.223 mS cm^−1^ after prolonged immersion and enabling “SOS”-style underwater communication through finger motion (Figure 7C(i–iii)). Beyond functional integration, long-term environmental adaptability has become an important criterion for evaluating underwater anti-swelling systems. Feng et al. [143] developed a PVP/P(HEA-AA) organohydrogel sensor using a PVP–DMSO/H_2_O binary solvent system to suppress water invasion, reducing equilibrium swelling from 43.1% to 9.4%. The hydrogel maintained structural and mechanical stability after 15 days under different pH conditions and enabled long-term monitoring of elbow, knee, and biomimetic whale-tail motions (Figure 7D(i–iii)). Likewise, Jia et al. [144] constructed a PPy/CMC/SA/PVA organohydrogel that exhibited only ~5% mass variation after 150 h immersion across multiple aqueous environments while preserving a mechanical strength of ~950 kPa and stable electromagnetic shielding performance, enabling reliable long-term underwater sensing. Overall, introducing anti-swelling characteristics has substantially improved the long-term stability and operational reliability of hydrogel sensors under complex underwater environments, providing an important foundation for future underwater intelligent sensing applications. However, the performance of anti-swelling hydrogels for underwater applications is determined not only by their ability to suppress swelling but also by the long-term retention of structural integrity, conductive networks, and sensing functionality during prolonged immersion. Therefore, for underwater motion-monitoring applications, performance evaluation should emphasize the simulation of realistic service environments rather than relying solely on static immersion tests. A comprehensive evaluation framework should incorporate different aqueous media, including deionized water, PBS, and artificial seawater, together with prolonged immersion periods (24 h, 7 d, 15 d, and 30 d), as well as environmental factors such as fluid flow, hydrostatic pressure, and salinity variations. Furthermore, dynamic testing under representative underwater exercise conditions, including swimming, repetitive joint motion, and continuous mechanical deformation, should be performed to comprehensively evaluate swelling behavior, mechanical retention, conductivity stability, wireless communication capability, and long-term signal reliability. Such a framework would provide a more realistic assessment of the long-term motion-monitoring capability of anti-swelling hydrogels under complex underwater environments.

Overall, anti-swelling hydrogels have demonstrated considerable potential for wearable sensing applications under high-humidity perspiration and underwater motion environments. Their key advantage lies in maintaining network integrity, reliable interfacial contact, and continuous conductive pathways, thereby mitigating the adverse effects of water uptake, interfacial hydration, and fluid disturbance on sensing performance. Nevertheless, current studies have largely focused on single environmental conditions or short-term validation, while the stability of anti-swelling hydrogels under the coupled effects of perspiration infiltration, ionic interference, continuous motion, fluid flow, and long-term wear remains insufficiently evaluated. Future efforts should therefore establish evaluation systems that more closely simulate realistic motion scenarios, enabling synergistic optimization of structural stability, interfacial reliability, conductivity retention, and signal accuracy, thereby promoting the transition of anti-swelling hydrogels from laboratory-scale sensing demonstrations to long-term reliable applications in complex motion environments.

## 5. Conclusions and Perspectives

As wearable sensors continue to evolve from short-term signal acquisition toward long-term continuous monitoring under complex hydrated environments, the design objective of anti-swelling hydrogels has gradually shifted from merely suppressing swelling to the synergistic enhancement of long-term structural stability, interfacial stability, and signal reliability. This review systematically summarizes the structural design strategies and internal water environment regulation mechanisms of anti-swelling hydrogel-based wearable sensors. Although these strategies operate through distinct mechanisms, they all aim to restrict water transport, stabilize polymer networks, and maintain the continuity of conductive pathways, thereby synergistically improving long-term stability under complex hydrated environments. Representative studies have demonstrated that network-confinement, surface-hydrophobic, core–shell, and gradient structures can all reduce the equilibrium swelling ratio to below 5%, highlighting the significant advantages of structural engineering in enhancing dimensional stability and long-term serviceability. Meanwhile, material regulation further controls the balance between free and bound water while stabilizing conductive networks through mechanisms including ionic/coordination crosslinking, nanoconfinement, zwitterionic hydration, and solvation-mediated anti-water exchange, thereby extending anti-swelling design from simple dimensional control toward the integrated regulation of conductivity retention, interfacial stability, and long-term sensing reliability. Based on the above analyses, this review further establishes an analytical framework linking structural design, internal water environment regulation, adaptation to complex environments, and motion-monitoring applications. Unlike previous reviews that primarily focus on material composition, anti-swelling strategies, or sensing performance, this review takes the practical service requirements of wearable sensors as its central theme, systematically connecting anti-swelling mechanisms with the evolution of the internal water environment, long-term interfacial stability, and applications in complex hydrated environments. Furthermore, it reveals the intrinsic relationship between internal water environment regulation and the long-term retention of electrical conductivity, interfacial stability, and signal reliability, while proposing an evaluation framework for realistic motion environments. These insights provide a valuable reference for the horizontal comparison and standardized evaluation of different anti-swelling hydrogel systems.

Although anti-swelling hydrogels have achieved equilibrium swelling ratios below 5%, and in some cases even exhibited nearly non-swelling behavior over prolonged periods, several challenges remain before their widespread implementation in practical wearable devices and industrial applications. First, current strategies primarily suppress water penetration and polymer chain expansion through network confinement, hydrophobic barriers, core–shell architectures, gradient structures, and ionic/coordination crosslinking. However, excessive structural constraints often compromise material flexibility, ion transport, interfacial adaptability, and wearing comfort, making it difficult to simultaneously optimize anti-swelling performance, electrical conductivity, adhesion, and sensing responsiveness. Second, existing evaluation systems still rely predominantly on macroscopic indicators, such as equilibrium swelling ratio, mass variation, and mechanical retention, while a systematic understanding of water transport pathways, the dynamic evolution of free and bound water, polymer–water interactions, and their intrinsic relationship with conductive network stability remains lacking. Furthermore, practical motion environments involve the coupled effects of perspiration infiltration, ion diffusion, skin friction, cyclic deformation, temperature and humidity fluctuations, and underwater fluid flow. Nevertheless, current studies remain largely confined to static immersion, short-term cyclic tests, or single-environment evaluations, and standardized evaluation protocols that realistically simulate high-humidity perspiration, underwater motion, and long-term wearable applications are still lacking. Finally, issues including fabrication reproducibility, batch-to-batch consistency, production cost, quality control, long-term biosafety, and regulatory approval continue to represent major bottlenecks hindering the large-scale manufacturing and industrial translation of anti-swelling hydrogel-based wearable sensors.

To address these challenges, future research should shift from merely reducing the swelling ratio toward establishing quantitative correlations between anti-swelling behavior and long-term sensing performance, thereby enabling the synergistic optimization of anti-swelling capability and long-term sensing stability. Meanwhile, greater efforts should be devoted to the development of in situ characterization techniques and quantitative mechanistic studies of the dynamic evolution of free and bound water, water transport pathways, and interfacial behavior, with the aim of elucidating their coupling mechanisms with conductive network stability, interfacial impedance evolution, and long-term signal reliability. On this basis, standardized evaluation protocols covering realistic service environments, including high-humidity perspiration, prolonged underwater immersion, cyclic deformation, and long-term wear, should be established. Furthermore, the coordinated advancement of material design, scalable manufacturing, and intelligent sensing systems will facilitate the translation of anti-swelling strategies that have demonstrated effectiveness under representative complex environments, such as high-humidity perspiration and underwater conditions, into practical wearable applications. Such efforts are expected to accelerate the long-term reliable deployment of anti-swelling hydrogel-based wearable sensors while providing robust material platforms for sports health monitoring and wearable sensing under complex hydrated environments.

## Figures and Tables

**Table 1:** Performance characteristics, advantages, and limitations of different structural anti-swelling strategies.

Structural Strategy	Representative System	Advantages	Limitations	Fabrication Complexity	Applicable Scenarios	Refs.
Network-Confinement Structures	PVA/PSBMA–PA–Li_2_SO_4_	Equilibrium swelling ratio reduced to 4.0% (DI water) and 14.3% (high-salinity solution) after 14 d while maintaining an elongation at break of 633%, enabling simultaneous anti-swelling capability and network flexibility.	Anti-swelling performance relies on highly crosslinked double-network architectures and synergistic ionic interactions; network optimization is relatively complex, and excessive crosslinking may compromise flexibility and increase energy dissipation.	Moderate	Long-term humid environments; wearable sensors for underwater; flexible strain sensing	[38]
	MXene/SA/PAM	Swelling ratio reduced to 10.8% while retaining 99.5% resilience and a conductivity of 1.44 S m^−1^, achieving synergistic enhancement of anti-swelling performance, mechanical properties, and electrical conductivity.	Anti-swelling performance mainly depends on suppression of polymer chain expansion by dense crosslinked networks; excessive network densification may reduce flexibility and conformability.	High		[8]
	PVA/P(SBMA-co-HEMA)	Equilibrium swelling ratio maintained at only 9% after 30 d, together with a toughness of 518 kJ m^−3^, indicating excellent long-term anti-swelling capability and mechanical stability.	Anti-swelling behavior depends on network confinement and electrostatic regulation; conductivity may decrease under acidic conditions due to protonation-induced aggregation.	High		[39]
Surface-Hydrophobic Structures	Gr–PMAm	Hydrophobic surface layer effectively suppresses water permeation, resulting in swelling ratios below 0.2% in water, seawater, sweat, and acidic/alkaline media while maintaining stable underwater electrical conductivity and sensing performance.	Hydrophobic surfaces may reduce interfacial wettability, thereby affecting functional component integration.	Moderate	Underwater wearable sensing; wearable sensors; underwater communication	[40]
	PDMAEMA/SA@PEI–MPS	Hydrophobic surface layer effectively inhibits water permeation, maintaining an equilibrium swelling ratio of approximately 40% together with stable electrical conductivity and sensing performance.	Anti-swelling performance depends on the integrity of the hydrophobic barrier; repeated swelling–deswelling cycles may induce interfacial microcracks, limiting long-term stability.	Moderate		[41]
	PAA/CHI/A1–MXene	Equilibrium swelling ratio reduced to 3.8% while maintaining an adhesive strength of 388.1 kPa and excellent underwater adhesion, enabling simultaneous enhancement of anti-swelling performance and interfacial adhesion.	Anti-swelling performance relies on an intact hydrophobic barrier, requiring a balance between water resistance and underwater adhesion.	High		[42]
Core–Shell Structures	PVA/ SA@STA/SR	Silicone rubber shell effectively blocks water exchange, resulting in a swelling ratio of only 1.00 ± 0.21% after 7 d underwater while maintaining stable electrical conductivity and underwater sensing performance, thereby enabling synergistic enhancement of anti-swelling capability, water resistance, and functional stability.	Anti-swelling performance depends on the integrity of the shell layer; shell damage markedly reduces water resistance, while limited self-healing capability may compromise long-term stability.	Moderate	Underwater wearable sensors; implantable flexible bioelectronics; soft robotics	[43]
	CS/PACG /Fe^3+^	Fe^3+^-induced highly crosslinked rigid shell reduces the equilibrium swelling ratio to 5.8%; the hydrogel remains structurally stable after 198 d of immersion while maintaining a compressive modulus of 4.25 MPa, achieving simultaneous enhancement of long-term stability and mechanical performance.	Anti-swelling performance relies on a stable metal-coordination network; weakened coordination under alkaline conditions may reduce shell stability and induce swelling failure.	Low		[44]
	PDMS/ AAm, PDMS/PVA–DMSO, PDMS/SA–AAm	Dense PDMS shell completely encapsulates the hydrogel core, limiting the swelling ratio to 1.9% after 40 d while maintaining stable electrical conductivity and underwater sensing performance, providing long-term water resistance.	Anti-swelling performance depends on continuous and intact shell coverage; the core–shell configuration limits direct interfacial contact and imposes higher requirements for conformability and long-term durability after mechanical damage.	High		[45]
Gradient Structures	PAM/PAAm–Gelatin-SA	Spatially graded crosslinking enables continuous regulation of network density, resulting in a swelling ratio of only 2.7% after 15 d while maintaining a toughness of 1239.5 kJ m^−3^, achieving synergistic enhancement of anti-swelling performance and mechanical robustness.	Anti-swelling performance relies on precise construction of the gradient architecture; magnetic-field-assisted gradient formation and metal-coordination regulation increase fabrication complexity.	High	Underwater wearable sensors; saline environments; underwater bioinspired actuators	[46]
	Gelatin	Continuous electrolyte gradients effectively suppress swelling, reducing the swelling degree (SD) from 43 to 27 while improving long-term anti-swelling stability and maintaining favorable interfacial performance.	Anti-swelling performance depends on precise gradient regulation; gradient formation requires strict control of diffusion and reaction conditions, posing challenges for large-scale fabrication.	Moderate		[47]

**Table 2:** Performance characteristics and design features of different anti-swelling engineering strategies.

Regulation Mechanism	Representative System	Anti-Swelling Performance	Mechanical Properties	Electrical Conductivity	Self-Healing Capability	Adhesive Performance	Biocompa-tibility	Sensing Performance	Refs.
Ionic/Coordination Crosslinking– Mediated Densification Mechanism	Cellulose/Al^3+^/AgNWs	ESR: 12.31% (DI water); 11.85% (salt water).	Tensile strength: 2.26 MPa; compressive strength: 16.99 MPa; compressive toughness: 2.86 MJ m^−3^.	Conductivity: 0.04 S m^−1^.	—	—	Biodegradable within 22 d.	GF: 0.24 (0–35%) and 0.70 (35–65%); sensing range: 0–65%.	[83]
	P(AA-MEA)-CS-Fe	NSR: −11% (DI water).	Tensile strength: 0.462 MPa; elongation at break: 1199%; toughness: 2.01 MJ m^−3^.	Conductivity: 0.326 S m^−1^.	Self-healing efficiency: 97.22%.	—	L929 cell viability >97%.	GF: 1.58 (0–100%), 3.01 (100–200%), and 5.25 (200–400%); sensing range: 0–400%.	[84]
	PSG-Zr^4+^-CNT	ESR: 1% (DI water).	Tensile strength: 202 kPa; elongation at break: 632%; toughness: 0.93 MJ m^−3^.	Conductivity: 0.42 S m^−1^.	—	—	—	Pressure sensitivity: 0.425 kPa^−1^ (0–3 kPa) and 0.075 kPa^−1^ (3–50 kPa).	[85]
Nanoconfinement-Mediated Anti- Swelling Mechanism	Cellulose/P(SBMA-co-HEMA)	SR: 48% (DI water); 38% (seawater).	Tensile strength: 336 kPa; Young’s modulus: 2.74 MPa.	Conductivity: 1 S m^−1^.	—	—	—	GF: 2.33 (R^2^ = 0.994); sensing range: 3–12% strain and 300 Pa–6 kPa.	[86]
	P(AA-co-SBMA)/BC	SR: ≈11% (DI water).	Tensile strength: 145 kPa; elongation at break: 1304%; compressive strength: 375 kPa.	Conductivity: 5.13 S m^−1^.	—	Wood, 20.6 kPa; acrylic, 14.3kPa; rubber, 16.0 kPa.	—	GF: 0.95 (1–200%), 1.59 (200–600%), and 2.14 (600–800%); response/recovery time: 270/220 ms.	[87]
Zwitterionic Hydration-Mediated Anti-Swelling	PSBMA-BCN	SR: ≈144% (DI water, pH 3); ≈144% (DI water, pH 10).	Elongation at break: 520%; toughness: 34.9 kJ m^−3^.	Conductivity: 3.96 S m^−1^.	—	Porcine skin, 1.1 kPa.	No skin irritation after 12 h.	GF: 1.99 (<70%) and 2.88 (70–126%); sensing range: 0.5–100% strain; response time: 0.16 s.	[88]
	PSBMA/ CNTs	SR: ≈50% (DI water).	Compressive strength: 0.76 MPa; tensile strength: 26.8 kPa.	Conductivity: 0.059 S m^−1^.	Self-healing time: 30 s; 82% tensile recovery after 3 min.	Stable adhesion to glass, plastic, and wood.	—	GF: 2.22 (0–125%), 5.14 (125–225%), and 10.35 (225–300%);	[89]
	Gel/PSAA-Al^3+^	SR: ≈32% (DI water).	Tensile strength: 252.7 kPa; elongation at break: 1053.8%; toughness: 111.2 kJ m^−3^.	Conductivity: 6.38 mS cm^−1^.	—	Paper, 12.92 kPa; wood, 12.25 kPa;	Good cytocompatibility toward L929 cells.	GF: 0.83 (0–200%), 1.29 (200–600%), and 1.61 (600–1050%);	[90]
Solvation-Mediated Anti-Water-Exchange Mechanism	PVA/Gel/TA/MXene Organic Hydrogel (PGTM)	SR: ≈16.6% (DI water); ≈17% (0.5 M HCl); <0.5% (0.9 wt% NaCl).	Tensile strength: 3.32 MPa; elongation at break: 649%.	Conductivity: 1.4 S m^−1^.	—	—	No erythema or edema after skin irritation tests.	GF: 3.15; sensing range: 0.01–400% strain; pressure response/ recovery time: 74.2/50 s.	[91]
	AL_2_CNF/PAM/PVA-EG_4_	SR: ≈10% (DI water).	Tensile strength: 135.8 kPa; elongation at break: 300%.	Conductivity: 0.244 S m^−1^.	—	90° peel strength: 42 N m^−1^.	Good viability of NIH3T3 cells.	GF: 0.81 (0–100%), 0.92 (100–200%), and 0.98 (200–300%); response/ recovery time: 140/160 ms.	[92]
	PSA-DA/DES	ESR: ≈3% (water); ≈20% (artificial seawater).	Tensile strength: 0.25 MPa; elongation at break: 1600%; toughness: 1.46 MJ m^−3^.	Conductivity: 0.85 S m^−1^.	Self-healing efficiency: 94%.	Paper, 199 kPa; iron, 119 kPa.	—	GF: 2.20 (0–100%), 3.10 (100–280%), and 3.90 (280–500%).	[93]

Abbreviations: ESR, equilibrium swelling ratio; NSR, negative swelling ratio; SR, swelling ratio; GF, gauge factor; DI, deionized water.

## Data Availability

No new data were created or analyzed in this study. Data sharing is not applicable to this article.

## Abbreviations

The following abbreviations are used in this manuscript:

AA	Acrylic acid
AAm	Acrylamide
AgNWs	Silver nanowires
BC	Bacterial cellulose
CNTs	Carbon nanotubes
CS	Chitosan
DAC	Directional annealing casting
DES	Deep eutectic solvent
DI	Deionized water
DN	Double network
EG	Ethylene glycol
ESR	Equilibrium swelling ratio
GF	Gauge factor
GO	Graphene oxide
HEMA	2-Hydroxyethyl methacrylate
IPN	Interpenetrating polymer network
LMA	Lauryl methacrylate
MMT	Montmorillonite
MPS	3-Mercaptopropyltrimethoxysilane
MXene	Two-dimensional transition metal carbides/nitrides
NSR	Negative swelling ratio
PAA	Poly(acrylic acid)
PAAm	Polyacrylamide
PAM	Polyacrylamide
PBS	Phosphate-buffered saline
PDMAEMA	Poly(2-dimethylaminoethyl methacrylate)
PDMS	Polydimethylsiloxane
PEI	Polyethyleneimine
PNIPAM	Poly(N-isopropylacrylamide)
PSBMA	Poly(sulfobetaine methacrylate)
PVA	Poly(vinyl alcohol)
RH	Relative humidity
SA	Sodium alginate
SBMA	Sulfobetaine methacrylate
SR	Swelling ratio
TA	Tannic acid
